# Lineage tracing of Notch1-expressing cells in intestinal tumours reveals a distinct population of cancer stem cells

**DOI:** 10.1038/s41598-018-37301-3

**Published:** 2019-01-29

**Authors:** Larissa Mourao, Guillaume Jacquemin, Mathilde Huyghe, Wojciech J. Nawrocki, Naoual Menssouri, Nicolas Servant, Silvia Fre

**Affiliations:** 1 Institut Curie, PSL Research University, INSERM U934, CNRS UMR3215, F-75248 Paris, Cedex 05 France; 2 Sorbonne University, UPMC University of Paris VI, F-75005 Paris, France; 3 Present Address: Section of Molecular Cytology and Van Leeuwenhoek Centre for Advanced Microscopy, Swammerdam Institute for Life Sciences, University of Amsterdam, Amsterdam, The Netherlands; 4 Vrije Universiteit Amsterdam, Department of Physics and Astronomy, De Boelelaan 1081, 1081HV Amsterdam, The Netherlands; 5 Institut Curie, PSL Research University, INSERM U900, 75005 Paris, France; 6 Mines ParisTech, PSL Research University, CBIO-Centre for Computational Biology, 75006 Paris, France

## Abstract

Colon tumours are hierarchically organized and contain multipotent self-renewing cells, called Cancer Stem Cells (CSCs). We have previously shown that the Notch1 receptor is expressed in Intestinal Stem Cells (ISCs); given the critical role played by Notch signalling in promoting intestinal tumourigenesis, we explored Notch1 expression in tumours. Combining lineage tracing in two tumour models with transcriptomic analyses, we found that Notch1+ tumour cells are undifferentiated, proliferative and capable of indefinite self-renewal and of generating a heterogeneous clonal progeny. Molecularly, the transcriptional signature of Notch1+ tumour cells highly correlates with ISCs, suggestive of their origin from normal crypt cells. Surprisingly, Notch1+ expression labels a subset of CSCs that shows reduced levels of Lgr5, a reported CSCs marker. The existence of distinct stem cell populations within intestinal tumours highlights the necessity of better understanding their hierarchy and behaviour, to identify the correct cellular targets for therapy.

## Introduction

Intestinal crypts have been reported to harbour two distinct types of stem cells: homeostatic stem cells, marked by the G-protein coupled receptor Lgr5^1^, that continuously generate new progenitors to ensure efficient renewal of the intestinal mucosa, and presumably quiescent stem cells, thought to provide a reserve source of stem cells that can be activated upon injury^2,3^. We have shown that the Notch1 receptor is expressed in both homeostatic and reserve stem cells populations *in vivo*, providing a valuable tool to mark both cell types and dissect their hierarchical relationship^4^. Intestinal tumours have been proposed to originate from intestinal stem cells (ISCs) or very early progenitors, as these are the only cells that persist long enough within the tissue to ensure clonal expansion of a mutant progeny^5^. However, differentiated cells and/or intestinal progenitors have also been shown to be able to undergo dedifferentiation upon activation of specific pathways and to acquire stem cell properties that result in tumour formation^6^. Nonetheless, these studies are based on genetic targeting of specific oncogenic mutations, while direct evidence showing the presence of CSCs within spontaneously arising intestinal tumours is still poor. Recently, a lineage tracing study in intestinal and colonic tumours using two stem cell-specific promoters, Lgr5 and Bmi1^7^, proposed that these two ISCs markers can be used to detect different stem cell populations in intestinal tumours^8^. We have previously shown that the specific expression of the Notch1 receptor in ISCs promotes active Notch signalling in these cells^4^ and, consistently with its essential role in intestinal homeostasis and cell fate determination^9^, Notch activity has been reported as essential for crypt stem cells maintenance^10^. Of relevance, Notch signalling is required for intestinal tumour formation and it cooperates with the Wnt pathway to promote crypt hyperplasia^11,12^. On these premises, we assessed whether the expression of the Notch1 receptor labels CSCs in intestinal and colon adenomas derived from both a genetic mouse model and a carcinogenic protocol. We performed clonal analysis using the Notch1-Cre^ERT2^ mouse line, which we have previously shown to mark multipotent ISCs in the small intestine and colon^4^, and assessed the identity and fate of Notch1+ cells within spontaneously arising intestinal tumours.

We show that Notch1-labeled cells represent a distinct population of CSCs within both intestinal and colon tumours, which contributes to tumour growth by clonal expansion and generates intra-tumoural heterogeneity. These *in vivo* studies provide evidence for the existence of different types of CSCs in intestinal tumours, which might have different origins and/or exhibit differential response to treatment.

## Results

### Notch1-Cre^ERT2^ labels undifferentiated and proliferative tumour cells

**Figure 1:**
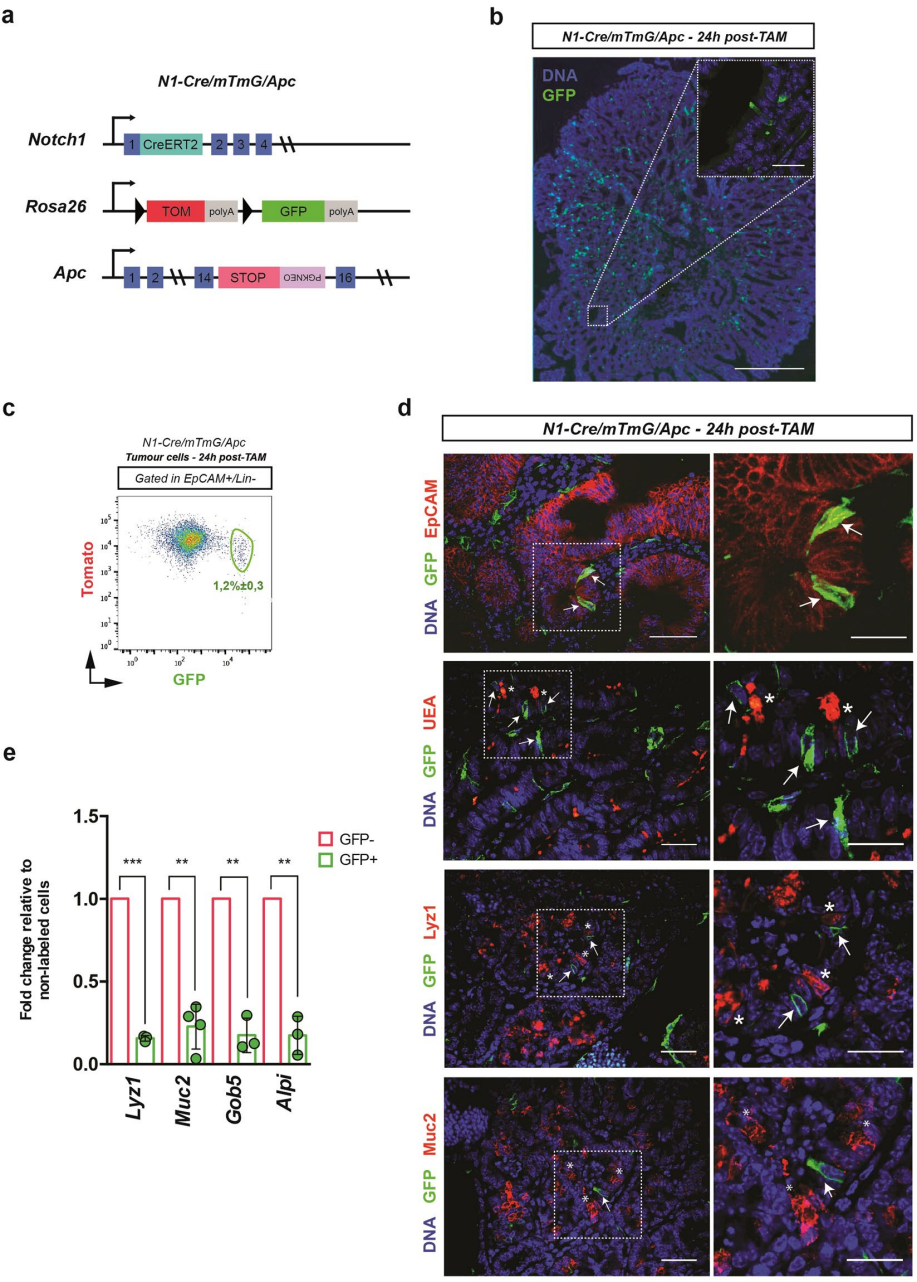
Notch1-Cre^ERT2^ labels undifferentiated and proliferative tumour cells. (**a**) Schematic representation of the triple transgenic mouse model used in this study. Notch1Cre^ERT2^ knock-in mice (referred to as N1-Cre) were crossed to Rosa26^mTmG^ reporter mice and to Apc^+/1638N^ mice (termed Apc). (**b**) Representative section of an intestinal tumour from N1-Cre/R26^mTmG^/Apc mice, 24 h post tamoxifen injection. The inset shows a higher magnification of a Notch1-expressing tumour cell (marked by GFP in green). DNA is labelled by DAPI in blue. Scale bars represent 200 μm and 15 µm in the magnification panel. (**c**) FACS analysis (see Supplementary Fig. 1 for gate strategy details) of tumour cells dissociated from N1-Cre/R26^mTmG^/Apc mice 24 h post induction. Lin+ cells were excluded and single epithelial tumour cells were gated as epithelial cells (Epcam+/Lin−), allowing the quantification of Notch1+ tumour cells. Note that GFP+ cells also display Tomato fluorescence 24 h after induction (GFP+/Tom+), as the Tomato protein is still present at this time point, even if recombination has occurred. (**d**) Immunofluorescence analysis of N1-Cre/R26^mTmG^/Apc tumour sections using anti-EpCAM, Agglutinin (UEA), anti-lysozyme (Lyz1) and anti-Mucin2, all in red. Notch1-expressing tumour cells are labelled in green (GFP+) and DNA is marked by DAPI in blue. Magnifications insets are shown in the right panels. Arrows indicate Notch1-expressing tumour cells and asterisks show secretory tumour cells. (**e**) qRT-PCR showing the relative RNA expression of Lyz1, Muc2, Gob5 and Alpi in Notch1+ (green bars) and non-recombined tumour cells (red bars). Bars represent the average ± SDs of independent biological replicates (n ≥ 3) normalized to the 18 S housekeeping gene. **P ≤ 0.005; *P ≤ 0.05. The p-values were calculated using the Paired Ratio t-test. Scale bars correspond to 15 µm in the inset in (**b**), 30 µm in (**d**) and 20 µm in the insets in (**d**).

To track Notch1+ intestinal adenoma cells *in vivo*, we generated a triple transgenic mouse (hereafter referred to as N1-Cre/R26^mTmG^/Apc) by crossing mice carrying both the Notch1Cre^ERT2^ (N1-Cre)^4^ and the Rosa26^mTmG^ (R26^mTmG^)^13^ reporter with Apc^+/1638N^ (*Apc*) mutant mice^14^ (Fig. 1a). Apc mice harbour a heterozygous germline mutation in the *Apc* tumour suppressor gene and spontaneously develop intestinal adenomas, initially detectable at around six months of age, due to loss of heterozygosity (LOH) at the *Apc* locus. In the compound N1-Cre/R26^mTmG^/Apc mice, the membrane-associated red fluorescent protein (mT) is expressed in all cells, while membrane-associated GFP (mG) marks Cre-targeted cells. To identify the cells expressing the Notch1 receptor within tumours, N1-Cre/R26^mTmG^/Apc tumour-bearing mice received a single dose of tamoxifen and were analysed 24 h later (Fig. 1b). Quantification by flow cytometry of the proportion of Notch1+ cells within tumour epithelial cells (selected with the markers EpCAM+/Lin-, see gate strategies in Supplementary Fig. 1), indicated, in agreement with our immunofluorescence results, that Notch1-expressing epithelial cells represent a rare tumour cell population comprising 1,2% ± 0,3% of tumour cells (Fig. 1c). It should be noted that, as the N1-Cre line also labels other types of stromal cells, we exclusively focused our analysis on epithelial cells, expressing the epithelial marker EpCAM (Epithelial cell adhesion molecule^15^) (Fig. 1d). Since *Apc* mutant intestinal tumours present differentiated tumour cells, we evaluated if Notch1 is expressed in such cells by immunostaining for differentiation markers for secretory cells, such as Agglutinin (Ulex Europaeus Agglutinin, labelling both Paneth and Goblet cells), Lysozyme1^16^ (a specific marker of Paneth cells) and Mucin2^17^ (expressed in Goblet cells) (Fig. 1d). None of these markers was expressed in GFP+ cells, consistently with the lack of Notch1 expression in secretory cells in the normal intestinal epithelium^9^. We also assessed the expression of secretory and enterocyte (alkaline phosphatase intestinal, Alpi^18^) markers by qRT-PCR on sorted tumour cells and confirmed that GFP+ cells show strongly reduced levels of expression for all of these markers (Fig. 1e), indicating that the N1-Cre mouse line labels undifferentiated tumour cells.

### Notch1 expression defines multipotent tumour cells with self-renewal capacity

**Figure 2:**
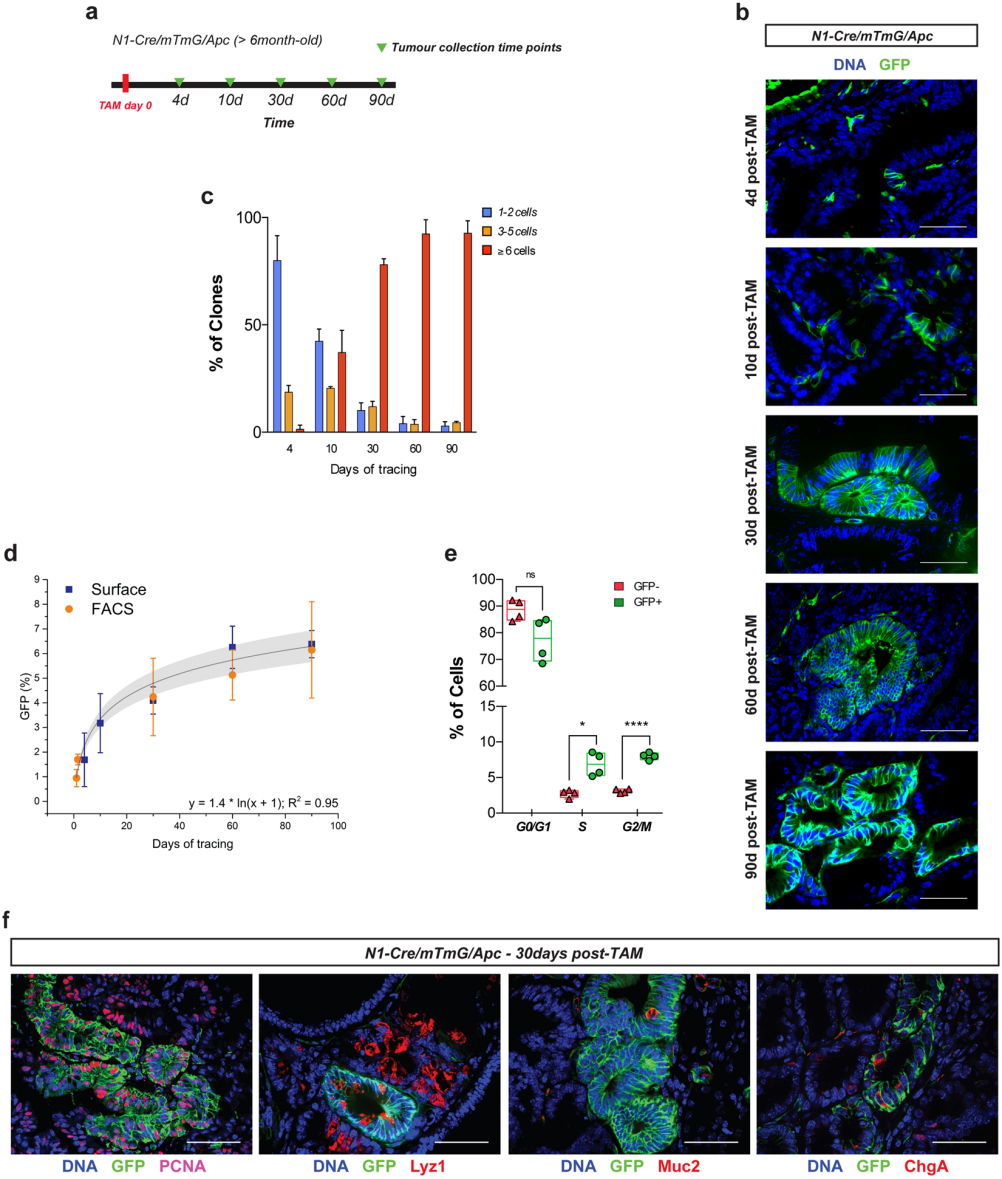
Notch1 expression defines multipotent tumour cells with self-renewal capacity. (**a**) Experimental timeline used to perform lineage tracing analysis in N1-Cre/R26^mTmG^/Apc tumours *in vivo*. Tumour-bearing mice were induced at the age of about 6 months and then culled at 4, 10, 21, 30, 60 and 90 days. (**b**) Representative images of N1-Cre/R26^mTmG^/Apc tumour sections after 4, 10, 30, 60 and 90 days of tracing, showing the rapid generation of Notch1+ clones that grow over time. Notch1-derived lineages are labelled in green (GFP+) and DNA in blue by DAPI. (**c**) Quantification of the rate of expansion of GFP+ cells in growing tumours. Mean (+/−SD) percentage of clones over the total number of clones counted per sample. Small clones (1–2 cells, in blue), intermediate clones of three to five cells (in orange) and large clones of more than six cells (in red) were counted. (**d**) Clonal analysis of Notch1+ tumour cells using a dual methodology (FACS and Surface) shows the growing capacity of Notch1+ tumour cells. Notch1-expressing GFP+ tumour cells were quantified by FACS at the following tumour collection time points: 24 h, 36 h, 30d, 60d and 90d (orange dots; at least three biological replicates per time point). Quantification of the GFP+ tumour area within the total tumour surface (see also Supplementary Fig. 2c) was carried out at 4d, 10d, 30d, 60d and 90d post tamoxifen induction (blue squares). Both quantifications were best fitted by the y = 1.4*ln(x + 1) logarithmic function (R^2^ = 0.95). (**e**) FACS quantification of non-dividing (G0/G1) and dividing (S and G2/M) tumour epithelial cells. Notch1+ tumour cells are represented by green dots and non-labelled cells by red dots. Boxes represent min to max values ± SDs of independent biological replicates (n = 4) ****P ≤ 0.0005; **P ≤ 0.005, *P ≤ 0.05, using paired t-test. (**f**) Immunofluorescence analysis of tumour sections showing Notch1-derived lineages (GFP, in green) 30 days after induction using anti-PCNA, anti-lysozyme (Lyz1), anti-Mucin2 and anti-Chromogranin A, all in red. DNA is marked by DAPI in blue. Scale bars correspond to 50 µm.

To map the fate of Notch1+ tumour cells and establish their self-renewal capacity *in vivo*, we examined tumour-bearing N1-Cre/R26^mTmG^/Apc mice at different time points after administration of tamoxifen, from 4 days up to 90 days (Fig. 2a). Our experimental approach required that we started tracing in tumour-bearing mice (thus in 6-month old animals, which generally succumb 2 to 3 months later) and consequently, a 90 days chase was the longest chase period for clonal analysis. Lineage tracing showed that Notch1+ tumour cells rapidly generate clones of marked progeny, visible already 4 days after labelling, and that these clones enlarge over time (Fig. 2b). To assess if GFP-labelled cells have progenitor properties, we analysed the rate of their clonal expansion in growing tumours using three different methods: by manual counting, we quantified the percentage of small, intermediate and large clones traced at each chase time point (Fig. 2c); by FACS analysis, we assessed the percentage of GFP+ cells within total tumour cells at the same time points (Supplementary Fig. 2a,b); by immunofluorescence, we computed the GFP+ tumour area within the total tumour surface, defined by the expression of the Tomato fluorescent protein (Supplementary Fig. 2c). A two-parameter logarithmic function yielded the best fit to describe both quantification methods. This function predicts that Notch1+ lineages will persist in adenomas and continue to slowly expand even after 90 days, indicating self-renewal capacity (Fig. 2d). In addition, Notch1+ tumour cells appear more proliferative than non-marked tumour cells, as shown by the significantly increased proportion of GFP+ cells in the S and G2/M phases of the cell cycle (Fig. 2e). We next assessed the cellular composition of Notch-derived clones and found that Notch1+ tumour cells can generate the repertoire of different cell types found within intestinal adenomas, as Notch1 lineages contain both proliferative (marked by Proliferating Cell Nuclear Antigen, PCNA in Fig. 2f) and differentiated tumour cells (marked by Lyz1, Muc2 and ChgA in Fig. 2f). The longevity of Notch1-expressing tumour cells, along with their multipotency, establishes that Notch1 is expressed *in vivo* in CSC.

### The transcriptional signature of Notch1+ tumour cells reveals a close correlation with the gene expression profile of normal ISCs

**Figure 3:**
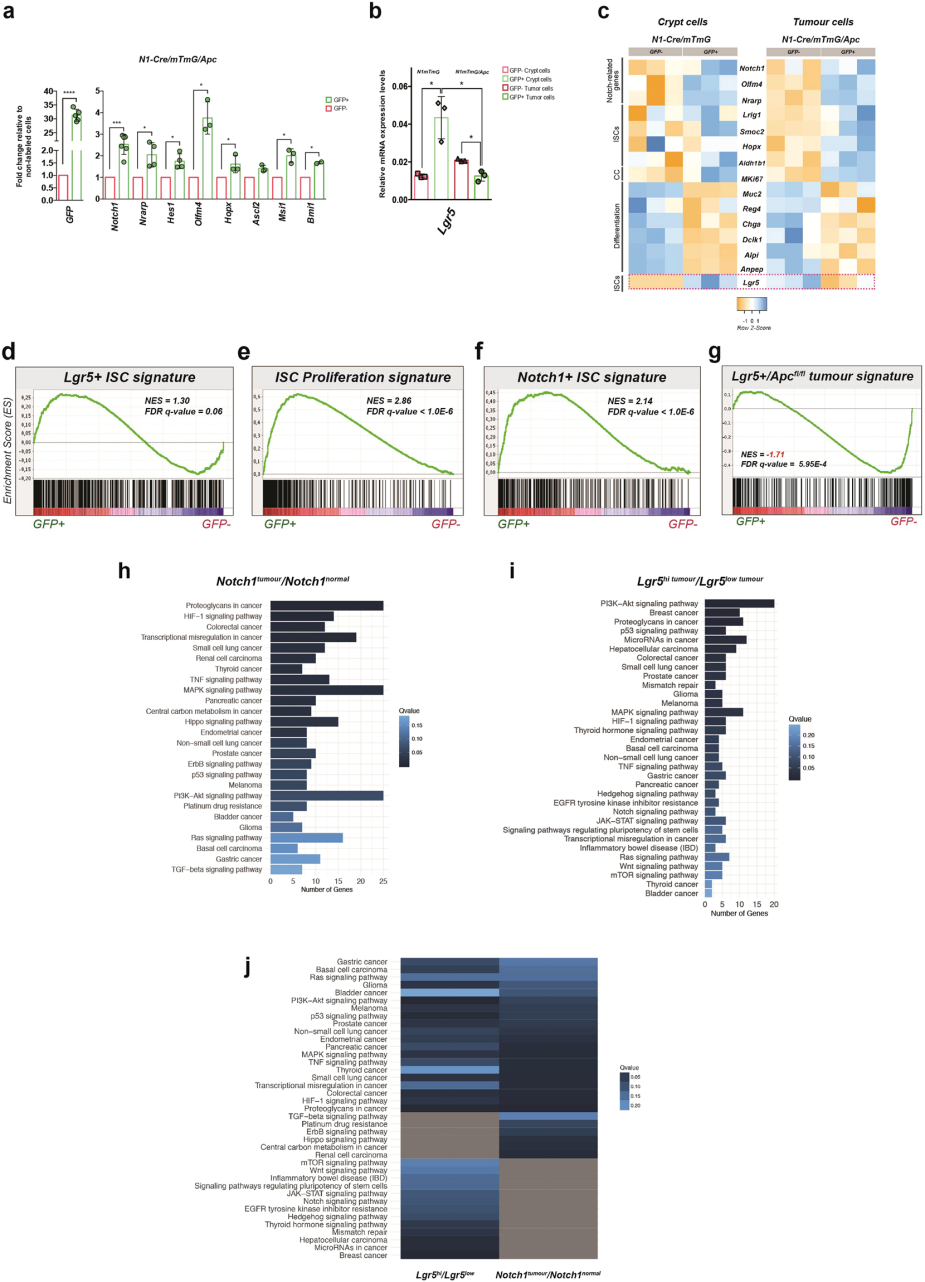
The transcriptional signature of Notch1+ tumour cells reveals a close correlation with the gene expression profile of normal ISCs. (**a**) RT-PCR for the indicated genes of sorted Notch1+ tumour cells (green bars) relative to non-recombined tumour cells (red bars). Data is expressed as the mean ± SDs of different biological replicates (each represented by dots) normalized by 18S housekeeping gene expression. ****P ≤ 0.0005, **P ≤ 0.005; *P ≤ 0.05. The p-values were calculated using the Paired Ratio t-test. (**b**) **q**RT-PCR showing the relative RNA expression levels of Lgr5 in Notch1+ crypt cells (GFP+ sorted cells from N1-Cre/R26^mTmG^ mice, in light green) and Notch1+ tumour cells (GFP+ sorted cells from N1-Cre/ R26^mTmG^/Apc mice, in dark green) relative to non-labelled cells (shown in light pink and dark pink). Data is shown as the average ± SDs of three independent biological replicates normalized by 18S housekeeping gene. *P ≤ 0.05 using the Student’s t-test (Welch correction). (**c**) Heatmaps for the expression patterns of selected genes extracted from genome-wide transcriptional datasets comparing Notch1+ crypt cells (GFP+ in N1-Cre/ R26^mTmG^ mice) and Notch1+ tumour cells (GFP+ in N1-Cre/ R26^mTmG^/Apc mice) relatively to non-labelled sorted crypt and tumour cells (GFP−). Expression levels of three independent biological replicates for each selected gene; Notch1, Notch1 targets (Nrarp, Hes1 and Olfm4), ISCs markers (Lrig1, Smoc2, Hopx, Aldh1b1 and Lgr5), proliferation antigen (MKi67) and differentiation markers (Muc2, Reg4, ChgA, Dclk1, Alpi and Anpep) are depicted by a scale of colour intensity measured by the Z-score (varying from −1 to 1). (**d–f**) Gene set enrichment analyses (GSEA) showing the enrichment plots between Notch1-expressing tumour cells and normal Lgr5+ ISC signature (**d**), ISC proliferative signature (**e**), and Notch1+ ISC signature (**f**). (**g**) Gene set enrichment analysis (GSEA) graph representing an inverse correlation between Notch1+ tumour cells and Lgr5+/Apc^fl/fl^ tumour cells. NES: Normalised Enrichment Score. KEGG pathway analysis of signalling pathways altered in Notch1-expressing tumour cells compared to Notch1+ ISCs. The graph displays selected signalling pathways associated with a qvalue <0.25. (**h–i**) KEGG pathway analysis of selected GO terms enriched in Notch1+ tumour cells vs Notch1+ normal cells (**h**) or in Lgr5^hi^ versus Lgr5^Low^ adenoma cells. All GO terms indicated presented a q value <0.25. (**j**) Comparison of GO terms enriched in the Notch1 and the Lgr5 tumour datasets, showing common and specific GO terms.

To molecularly characterise the tumour cells that express the Notch1+ receptor, we initially assessed the expression of selected genes in sorted cells by qRT-PCR. We confirmed that GFP+ cells expressed high levels of GFP, Notch1, Nrarp, Hes1 and Olfm4, the three-latter representing direct Notch targets^19–21^ (Fig. 3a), indicating that the Notch pathway is indeed active in Notch1+ tumour cells. Notch1+ tumour cells (GFP+) also showed enriched expression in reported markers of ISCs, such as Hopx^2^, Musashi1^22^ (Msi1) and Bmi1^7^ compared to GFP-epithelial cells within the same tumours (Fig. 3a). Next, we defined the genome-wide transcriptional signature of Notch1+ tumour cells and normal ISCs by performing Affymetrix analyses of FAC-sorted GFP+ and GFP- tumour and normal crypt cells derived from N1-Cre/R26^mTmG^/Apc and N1-Cre/R26^mTmG^ mice, respectively. This analysis confirmed that both tumour cells and normal ISCs expressing the Notch1 receptor are undifferentiated, as they show downregulation of differentiation markers, such as Mucin2 (Muc2), the Regenerating Family Member 4, Reg4^23^, Chromogranin A^24^ (Chga), Doublecortin Like Kinase 1 (Dclk1^25^), Alpi and Membrane Alanyl Aminopeptidase (Anpep^26^) both enterocyte markers, while they express high levels of reported ISCs markers, including Olfm4^27^, Lrig1^28,29^, Smoc2^30^, Hopx and Aldh1b1 (Aldehyde dehydrogenase 1b1^31^) (Fig. 3c). Unexpectedly, while in normal crypts the Notch1 and Lgr5 transcripts are enriched within the same cells, Notch1+ tumour cells showed a reduction in Lgr5 expression compared to non-labelled cells (Fig. 3b,c).

The expression profile of Notch1+ tumour cells highly correlates, by Gene Set Enrichment Analysis (GSEA), with published transcriptomic signatures defining normal ISCs^30^ (Fig. 3d) and their proliferative features^32^ (Fig. 3e). Consistently, we also found a tight correlation between the cells that express Notch1 in normal crypts and in tumours (Fig. 3f). These results suggest that the transcriptional landscape is not substantially changed between normal Notch1+ ISCs and CSCs, although we could detect a significant activation of cancer-related signatures (i.e. colorectal, gastric, pancreatic, lung, prostate cancer, basal and renal cell carcinoma) and several oncogenic signalling pathways (such as PI3K/Akt, p53, MAPK, TNF, TGF-beta, Ras, HIF-1, ErbB and Hippo signalling pathways) in Notch1+ tumour cells compared to Notch1+ normal ISCs (Fig. 3h and full KEGG analysis in Supplementary Table 1). Collectively, these results indicate that Notch1-expressing tumour cells are transcriptionally related to normal ISCs. Based on the comparison between these two transcriptional signatures, it is tempting to speculate that Notch1+ CSCs originate from ISCs.

### Notch1 and Lgr5 lineages are hierarchically organised

Given our surprising results regarding Lgr5 expression in Notch1+ cells (Fig. 3b,c), we then compared the signature of Notch1+ tumour cells to the transcriptome of Lgr5+ tumour cells^33^ and found an inverse correlation by GSEA, suggesting major differences between these two tumour cell populations (Fig. 3g). To further explore these differences, we have compared the main GO terms that characterize the signatures of Notch1+ tumour cells to the one generated by Schepers *et al*. derived from Apc-mutant Lgr5+ tumour cells (Fig. 3h–j). As indicated in Fig. 3j, most cancer-related signatures and several oncogenic pathways are enriched in both cell populations. However, thanks to this comparative exploration, we could identify pathways specifically activated in the Notch1+ tumour population, such as TGF-beta, ErbB and Hippo signalling pathways, and others that result statistically different only in Lgr5^hi^ adenoma cells, i.e. JAK/STAT, mTOR, Hedgehog signalling. All GO terms and their corresponding gene IDs, including a full list of genes that can be used to distinguish both Notch1+ and Lgr5^hi^ tumour cells are indicated in Supplementary Table 1.

It should be noted that the Notch pathway results upregulated in the Lgr5^hi^ tumour signature and not in our Notch1 dataset, since we have compared deregulated genes between Notch1+ normal crypt cells and Notch1+ tumour cells, both presenting activation of Notch signalling. Similarly, Wnt signalling is activated in the Lgr5^hi^ dataset, as expected from a list of genes enriched in Lgr5^hi^ tumour cells, where constitutive Wnt activation (*Apc* loss) has been targeted^33^. However, the Wnt pathway is not significantly increased in the Notch1+ signature, suggesting that Notch1+ tumour cells do not present higher Wnt activity than Notch1+ ISCs and in agreement with reduced Lgr5 expression in these cells (Fig. 3b,c).

**Figure 4:**
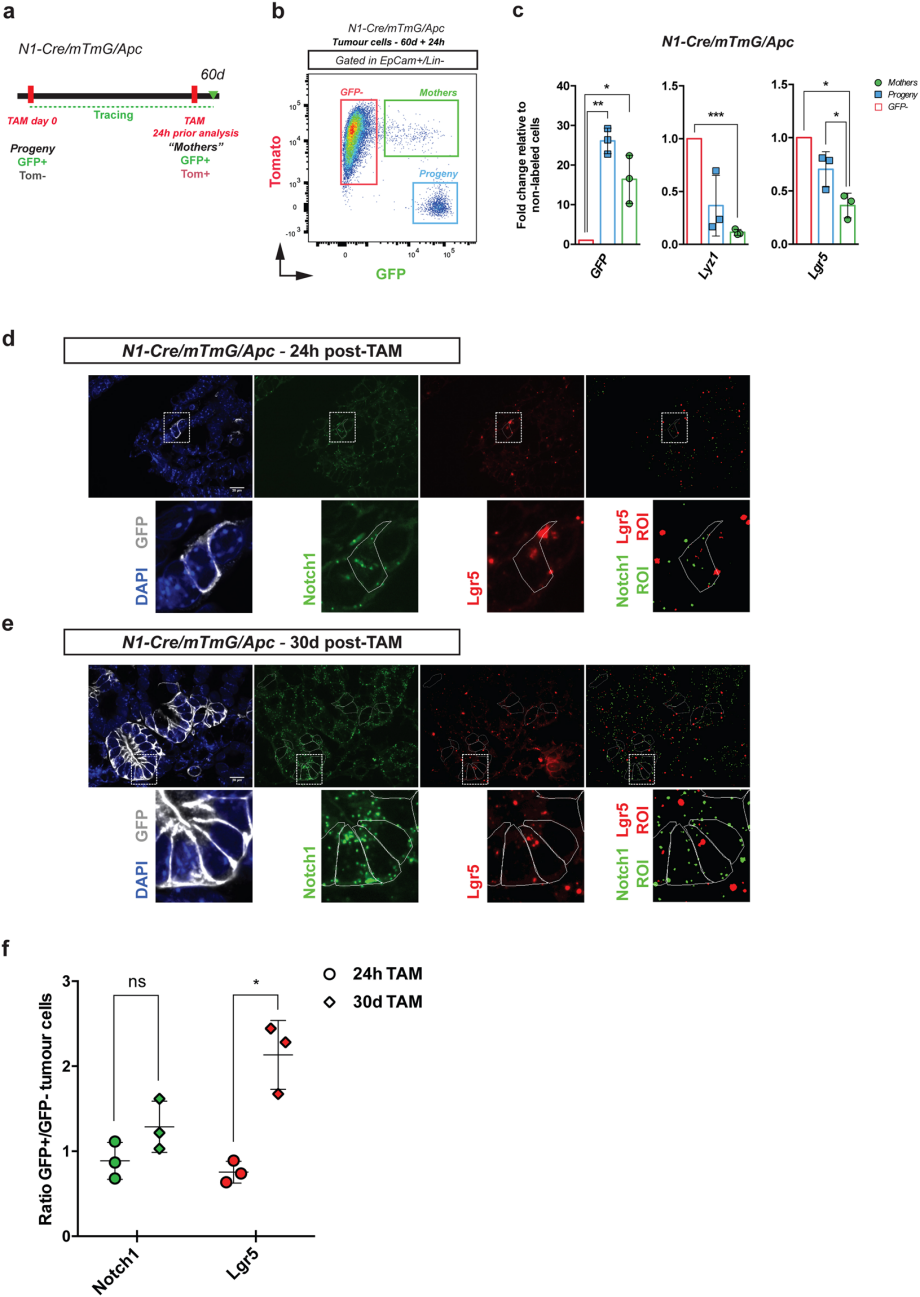
Notch1 and Lgr5 lineages are hierarchically organised. (**a**) Schematic representation of the tamoxifen administration protocol used to label Notch1-expressing cells (“Mothers”) and Notch1-derived progeny within the same mouse. N1-Cre/ R26^mTmG^/Apc tumour-bearing mice were induced with a single pulse of tamoxifen at day 0 and allowed to generate a clonal progeny for 60d (Progeny; GFP+/Tom- cells). 24 h before culling, mice received a second injection of tamoxifen, in order to label Notch1-expressing tumour cells (GFP+/Tom+cells). (**b**) Representative FACS dot plot of N1-Cre/ R26^mTmG^/Apc tumour cells analysed upon a 60 days chase and a 24 h re-pulse. Dissociated tumour cells were gated as epithelial cells (Epcam+/Lin−) and Notch1+ tumour cells (“Mothers” gated in green; GFP+/Tom+), Notch1 progeny (GFP+/Tom− cells, in blue) and non-labelled tumour cells (GFP−/Tom+, in red) were analysed. (**c**) RT-PCR analysis showing mRNA expression levels of GFP, Lysozyme1 (Lyz1) and Lgr5 in Notch1-expressing tumour cells (Mothers represented by green bars) and Notch1-derived Progeny (Progeny displayed in blue), relative to GFP− cells (in red). mRNA expression was normalized to 18S. Data is expressed as the mean ± SDs of biological replicates (n = 3) normalized by 18S housekeeping gene expression. ***P ≤ 0.005; **P ≤ 0.005; *P ≤ 0.05. The p-values were calculated using the Paired Ratio t-test. (**d**,**e**) Representative sections of N1Cre/R26^mTmG^/Apc tumours analysed either 24 h (**d**) or 30 days (**e**) post tamoxifen induction by RNAscope *in situ* hybridisation: split channel panels represent from left to right: DAPI (blue) and immunofluorescence anti-GFP (to visualise N1Cre-labelled tumour cells) in white; smFISH for Notch1 (green punctate dots) or Lgr5 (red punctate dots) and segmented and processed image representing the ROI that were automatically counted. Scale bars correspond to 20 µm. (**f**) Quantification of the number of mRNA fluorescent probe signals for Notch1 (green) or Lgr5 (red) in GFP+/GFP-negative cells at 24 h and 30d post tamoxifen induction. Data is shown as the individual ratio of three biological replicates (n = 3 tumours analysed per time point) ± standard deviation. *P ≤ 0.05 using the Welch t-test.

To investigate the hierarchy between these different tumour cell populations, marked by Notch1 or Lgr5 expression, we used the R26^mTmG^ double fluorescent line, allowing us to distinguish between initially labelled cells (that we called “Mothers”) and their derived progeny. Notch1-expressing cells were isolated 24 h after tamoxifen administration; within such a short chase, Notch1+ cells have turned green (GFP+) but retain the expression of the Tomato protein, due to protein stability, so they are GFP+ and Tom +. By labelling Notch lineages in tumours during a 60 days chase and giving a second tamoxifen pulse 24 h prior to tumour dissection (Fig. 4a), we can distinguish the recently labelled cells (“Mothers”, expressing both GFP and Tomato) from their progeny (GFP+ but Tomato-negative) and from non-labelled cells (GFP-negative) (Fig. 4b). qRT-PCR analysis of these three FACS sorted populations showed that Notch1-derived lineages (in blue) express intermediate levels of both Lgr5 and differentiation markers, such as lysozyme, between non-labelled cells (in red) and Notch1-expressing tumour cells (in green) (Fig. 4c). These results are consistent with our clonal analysis, suggesting that multipotent Notch1+ tumour cells can give rise to heterogeneous lineages comprising distinct cell types, such as differentiated cells (Lyz1+), and raise the intriguing possibility that Notch1+ tumour cells might generate Lgr5+ tumour cells, thus contributing to tumour growth and propagation. To test this hypothesis, we performed single molecule Fluorescent *in situ* hybridisation (smFISH) analysis of the expression of Notch1 and Lgr5 in both Notch1-expressing cells (24 h upon tamoxifen) and in clones derived from Notch1-targeted cells (1-month chase) (Fig. 4d,f). Confirming our transcriptomic data, we found that Notch1+ tumour cells express lower levels of Lgr5 than non-labelled tumour cells, as the ratio GFP+ /GFP- cells is less than 1. Moreover, the results upon long-term tracing provide additional evidence supporting our hypothesis that Notch1+ cells give rise to Lgr5+ daughter cells, as the ratio of the number of punctate dots between GFP+ /GFP- is around 2 in the clones derived from Notch1-expressing cells, even if the levels of Notch1 remain stable, confirming self-renewal capacity. It follows that Lgr5 is expressed at higher levels in GFP+ progeny (Fig. 4c and 30d in Fig. 4f) than in Notch1-expressing cells (“Mothers” in Fig. 4c and 24 h in Fig. 4f).

### Clonal analysis of Notch1+ tumour cells in chemically induced colon tumours

**Figure 5:**
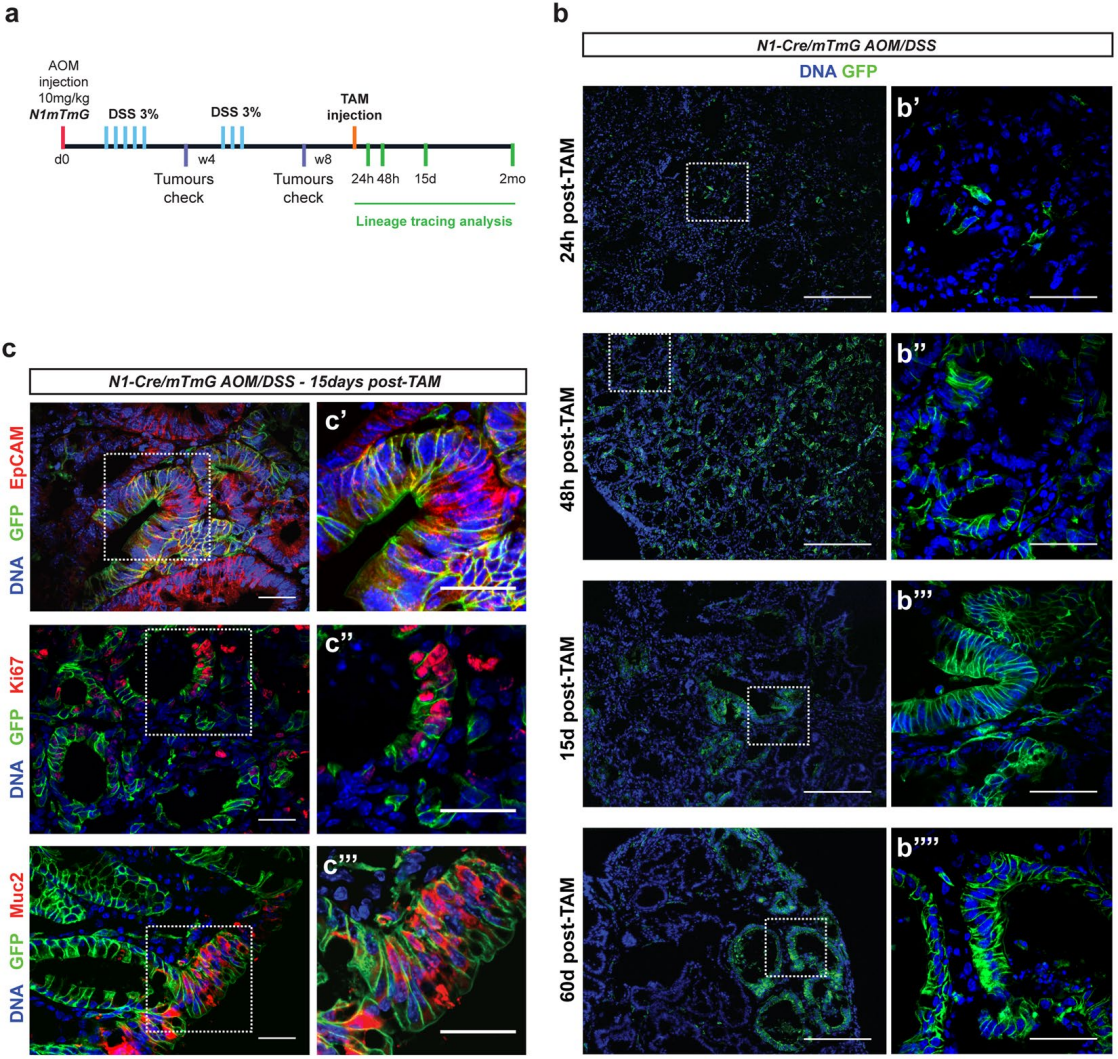
Clonal analysis of Notch1+ tumour cells in chemically induced colon tumours. (**a**) Experimental protocol and lineage tracing analysis in chemically induced colon tumours. N1-Cre/R26^mTmG^ mice were injected with AOM at day 0 (d0) and received 2 cycles of 3% DSS (blue bars), spaced by 3 weeks of recovery. After 4w (4 weeks) and 8w (8 weeks) the development of colon tumours in control mice was monitored by colonoscopy. Lineage tracing analysis was started after 8 weeks and tumours were harvested at the indicated time-points (green bars). (**b**) Representative sections of colon tumours analysed at the indicated time-points after tamoxifen administration. Notch1+ colon tumour cells and Notch1-derived clonal progeny present membrane GFP expression (in green). DNA is marked in blue with DAPI. (**c**) Immunostaining of tumour sections 15 days after Cre induction using anti-EpCAM, anti-Ki67 and anti-Mucin2 (all in red). Notch1+ cells are revealed by membrane GFP expression in green and nuclei in blue. Insets highlight areas of tumours containing clones derived from Notch1-expressing colon tumour cells that co-localize with the used markers. Scale bars represent 200 μm in (**b**), 50 µm in (b’–b””) and 30 μm in (c, c’–c”’).

Our clonal analysis in genetically modified mouse models indicated that the Notch1 receptor is expressed in a population of CSCs in intestinal adenomas. To further validate the clinical value of our study, we then examined if Notch1 expression could assist in identifying CSCs in chemically induced colon tumours, the most predominant location in colorectal cancer (CRC) patients. For these studies, we administered azoxymethane (AOM), followed by two cycles of exposure to the inflammatory agent dextran sodium sulphate (DSS)^34^ to N1-Cre/R26^mTmG^ mice. Upon colon tumour formation, which we monitored by colonoscopy^35^, we induced GFP expression with a single dose of tamoxifen and analysed the tumours at different chase times (24 h, 48 h, 15 days and 2 months) (Fig. 5a). The presence of uniformly scattered GFP+ cells 24 hours post-tamoxifen in all colon tumours analysed demonstrated that Notch1 is also expressed in chemically induced colon tumours (Fig. 5b). Consistently with our results in *Apc* mutant adenomas, Notch1+ colon tumour cells were also undifferentiated and proliferative, as they rapidly produced a clonal progeny, evident at 15 days after induction. A 2-month chase showed that Notch1-derived clones had considerably grown, suggesting that Notch1+ colon tumour cells self-renew, like their small intestinal counterparts (Fig. 5b).

To assess multipotency of the Notch1+ colon cancer cells, we analysed their clonal progeny (EpCAM+) and found that they express both proliferation (Ki67+) and differentiation markers (i.e. Mucin2 shown in Fig. 5c), demonstrating that Notch1-expressing cells in colon tumours have multilineage differentiation potential and present the same properties of Notch1+ intestinal adenoma cells. Notch1-expressing cells represent thus genuine CSCs in both genetic and carcinogenic tumour models. It remains uncertain however to which extent these cells contribute to the overall tumour development and growth.

## Discussion

Our study shows that Notch1+ expression labels a previously uncharacterised and distinct population of undifferentiated and self-renewing tumour cells, which clonally expand during tumour growth and generate heterogeneous lineages. Importantly, we found that Notch1+ tumour cells do not coincide with CSCs expressing the Lgr5 receptor, while they share a transcriptional signature with normal ISCs. In the normal intestine, two functionally distinct populations of ISCs have been defined^36^: rapidly dividing crypt-based columnar (CBCs) cells at the bottom of the crypt and “+4” ISCs, located at position +4 above the crypt base and considered as slower cycling, reserve stem cells. We have previously shown that Notch1 expression marks both types of ISCs^4^. The fact that Notch1+ tumour cells present an expression profile very similar to the signature of Notch1+ ISCs (Fig. 3f), prompts us to speculate that Notch1+ tumour cells might derive from +4 ISCs, shown to express lower levels of Lgr5 than CBCs and paramount for intestinal regeneration^3^.

**Figure 6:**
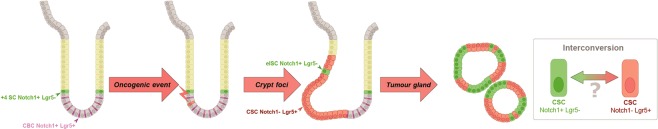
Proposed model for a cellular hierarchy within spontaneous tumours. In homeostatic conditions, Notch1 is expressed in both types of ISCs, the +4 SCs (in green), and the CBC ISCs, enriched in Lgr5 expression (in purple). According to the bottom-up model for tumourigenesis, an oncogenic hit, such as loss of the tumour suppressor *Apc*, should occur in an ISC to initiate crypt hyperplasia (crypt foci, in red). In such a scenario, Notch1+ normal ISCs become surrounded by mutant tumour-initiating cells (red cells) and thus get engulfed within the tumour. As mutant cells expand, their tumour microenvironment provides signals that potentiate the growth of these “embedded ISCs” (eISCs, in green), which express lower levels of Lgr5 relatively to normal ISCs and to the tumour bulk (see Fig. 3c). As the adenoma continues to develop over time, eISCs remain proliferative and multipotent within tumour glands, actively producing differentiated cells and generating new Notch1+ CSCs, but also Lgr5+ CSCs (red cells) that display lower levels of Notch1. The existence of two types of CSCs, one predominantly expressing Notch1 and the other Lgr5, might contribute to intra-tumoural heterogeneity, which drives tumour progression, by escaping to targeted therapies. Whether Lgr5+ CSCs are reciprocally able to produce/interconvert into Notch1+ CSCs remains to be established.

Unexpectedly, we found here an inverse correlation between the transcriptional profile of Notch1-expressing tumour cells and the signature of Lgr5^hi^ tumour cells published by Schepers and colleagues^33^. Such discrepancy might be due to the different models used in the two studies: Schepers *et al*. analysed Lgr5-expressing cells within intestinal tumours in which the homozygous deletion of the *Apc* gene has been specifically targeted to Lgr5+ cells. This approach greatly differs from ours, where we traced Notch1+ cells in spontaneously arising tumours generated by somatic loss of heterozygosis (LOH) at the *Apc* locus or by a carcinogenic agent. In these models, we found that Notch1+ tumour cells strikingly resemble wild-type ISCs, inferring that they might represent normal stem cells that were engulfed during tumour growth and thrive in the tumour environment thanks to paracrine mitogenic signals from surrounding cells (Fig. 6). Our work provides support to the original hypothesis that normal ISCs might contribute to tumour expansion and heterogeneity. Independently of their origin, the presence of wild-type ISCs within tumours and their possible contribution to tumour growth requires further investigation, due to the clear complications that it entails in developing new CSC-targeted drugs.

The comparison we report between Notch1+ and Lgr5^hi^ tumour signatures indicates that the Wnt pathway is not activated in Notch1+ tumour cells, as compared to normal ISCs. In agreement with this finding, activation of Notch signalling in *Apc* tumours has been shown to downregulate Wnt target genes^37^. Indeed, we have previously reported that Notch signalling promotes tumour initiation in synergy with Wnt, but at the same time impairs CRC progression by negatively regulating the transcription of Wnt target genes^11^.

Of relevance, our results define a cellular hierarchy within tumours, whereby Notch1+ CSCs appear to be giving rise to Lgr5+ tumour cells. Given the well-established plasticity and interconversion of different cell types in the normal intestine^2,3^, it is possible that Lgr5+ tumour cells could also generate Notch1+ cells, though only Lgr5 clonal analysis in the same mouse model could unambiguously establish if this is the case. Similarly to normal crypts, several studies reported that intestinal tumours comprise distinct pools of functionally different CSCs^38–42^, that can reciprocally and dynamically interconvert, corroborating our results on the existence of two CSCs populations in colorectal cancer, marked by Notch1 or Lgr5 (Fig. 6). In line with our results, Notch activity was shown to mark CRC tumour cells with low levels of Wnt activity and a pronounced epithelial phenotype^41^. This recent work provides evidence that different populations of CSCs could be distinguished by examining the Notch and Wnt activation within tumours. The transcriptomic signatures obtained here may provide the means to identify novel biomarkers to distinguish normal stem cells and tumour cell subpopulations with high Wnt or Notch activities.

CSCs plasticity in colon cancer has been well documented; as a consequence, targeted ablation of Lgr5+ cells is not sufficient to eradicate tumours as other cells can revert to Lgr5+ CSCs and contribute to tumour re-growth upon an initial regression^40,42,43^. In this study, we document the existence of distinct types of CSCs that can dynamically interconvert and we support the emerging concept that only combined targeting of several tumour cell populations could effectively counteract CSCs plasticity^39^.

Further functional studies will reveal the differences between the distinct CSCs populations here described, in particular regarding their respective clonogenic and metastatic potential. It will also be crucial to test their resistance to commonly used chemotherapeutic drugs, to possibly stratify patients based on their relative abundance.

## Methods

### Mice

Notch1Cre^ERT2^ ^4^ and Apc^+/1638^ ^14^ mouse lines were crossed to the dual fluorescent reporter Rosa26^mT/mG^ ^13^. GFP expression was induced in N1-Cre/R26^mTmG^/Apc 6–8 month-old mice or in N1-Cre/R26^mTmG^ adult mice by intraperitoneal injection of 0.1 mg/g of mouse body weight of tamoxifen free base (Euromedex). Mice were culled either 24 h post-induction (Notch1-expressing cells) or at different time points ranging from 36 h up to 3 months after induction. For each time point, at least three mice displaying tumours were analysed. No GFP fluorescence was observed in non-induced mice.

### Ethics Statement

All studies and procedures involving animals were in strict accordance with the European and National Regulation for the Protection of Vertebrate Animals used for Experimental and other Scientific Purposes. The project was approved by the French Ministry of Research with the references #04240.03 and APAFIS#8923-2017021515477911vi and was fully performed in the Animal Facility of Institut Curie (facility license #C75-05-18). We comply also with internationally established principles of replacement, reduction, and refinement in accordance with the Guide for the Care and Use of Laboratory Animals (NRC 2011). Husbandry, supply of animals, as well as maintenance and care of the animals in Exempt Of Pathogen Species (EOPS) environments before and during experiments fully satisfied the animal’s needs and welfare. Suffering of the animals has been kept to a minimum.

### Tissue dissociation into single cells

Freshly harvested tumours were dissected in DMEM/F12 (2% PS), followed by incubation in 5 mM EGTA-PBS at 4 °C, shaking gently, to remove potential contaminant cells from the normal tissue. Tumours were then minced in small pieces with razor blades and incubated in diluted TrypLE Express in PBS (66%), shaking (180 rpm) for 45 minutes at 37 °C. Trypsin was inactivated with 10% of cold FBS. The cell suspension obtained was then filtered through a 40 µm cell strainer and cells were counted upon centrifugation for 5 min at 450 g, following re-suspension in Flow buffer (DMEM/F12, 5 mM EDTA, 1% BSA, 1% FBS, 10U/ml DNAse (D4263-5VL Sigma-Aldrich)). Small intestines were harvested and flushed with cold 1X PBS, followed by longitudinal opening and cut into small pieces of approximately 5 mm × 5 mm. Intestinal fragments were subsequently incubated with 2 mM EDTA in HBSS for 30 minutes at 4 °C. Crypts were obtained by serial fractioning. Optimal crypt isolation was checked under the microscope. Crypts were then incubated in TrypLE Express (ThermoFisher Scientific) for 5 minutes at 37 °C to obtain single cells. TrypLE Express was inactivated with 10% cold FBS (Sigma-Aldrich). The cell suspension obtained was filtered through a 40 µm cell strainer and counted cells were resuspended in Flow buffer.

### Immunofluorescence

Freshly dissected intestines and tumours were washed in 1X PBS and fixed at room temperature with 4% PFA under agitation for 2 h. The samples were then impregnated with 30% Sucrose (for at least 24 h at 4 °C) or dehydrated in 70% Ethanol and embedded in OCT (VWR) or in paraffin, respectively. Samples were sectioned either in a cryostat or microtome at 5 µm thickness. For immunofluorescence staining, frozen sections were incubated with 0,3% Triton-blocking buffer (5% FBS and 2% BSA in PBS). Paraffin-embedded sections were rehydrated through a gradient of Ethanol. Subsequently, antigen retrieval was achieved by boiling in antigen unmasking solution (H-3300 Vector laboratories) for 20 minutes for all antibodies. The following primary antibodies were used: chicken anti-GFP (1:800, Abcam ab13970), rabbit anti-Lysozyme (1:500, Dako A009902), rabbit anti-Chromogranin A (1:200, Immunostar 20086), rabbit anti-Mucin2 (1:100/200, Clone PH497), mouse anti-PCNA (1:1000, Abcam ab29), rabbit anti-Ki67 (1:200, ab15580), rabbit anti-EpCAM (1:400, Abcam, ab32392). Secondary antibodies were incubated in PBS for 1–2 hours at room temperature. The following secondary antibodies were used: anti-chicken AlexaFluor488 (1:500, Invitrogen A-11039), anti-rabbit AlexaFluor633 (1:500, Invitrogen A-21071), anti-mouse AlexaFluor633 (1:500, Invitrogen A-21202), anti-mouse Cy3 (1:500, Jackson laboratories 92557), anti-rabbit Cy3 (1:500, Jackson laboratories 91144). Identification of secretory cells on frozen sections was performed using Ulex Europaeus Agglutinin I (UEA) directly coupled to Rhodamine (1:50, Vector Laboratories RL-1062). DNA was stained with DAPI.

### Quantification of clonal expansion by immunofluorescence

Whole tumour 4–5 µm frozen sections from mice culled at different time points after tamoxifen injection were imaged for GFP (recombined tumour cells) and Tomato endogenous fluorescent proteins. To quantify the GFP surface area over the total tumour area, we used an unbiased approach taking advantage of the ImageJ Software threshold tool. By adjusting the threshold of each individual channel (GFP and Tomato), we extracted a binary image that contained exclusively black and white pixels and quantified these using a macro developed for ImageJ software. The sum of the Tomato pixels reflected the total area of a tumour (Σ pixels TOM; hence referred as A) and the sum of the GFP pixels indicated the recombined areas (Σ pixels GFP; called B). Since we have used homozygous mice carrying two copies of the Rosa26^mTmG^ allele, resulting in GFP+/Tomato+ cells, GFP+ areas were subtracted from total (Tomato+) area. The final area (A − B) was set to 100% and GFP area was calculated by the equation: GFP (%) = B*100%/(A − B).

### Fitting curve

The concatenated fit of the GFP cells expansion data from FACS and the surface-based methods was obtained using OriginLab software (OriginLab Inc., MA, USA). To account for the initial rapid increase and later slower growth of the GFP population, a logarithmic function has been chosen, and the quality of the fitting was judged by visual inspection and analysis of fit residuals and proved more appropriate than using an exponential decay function.

### smFISH RNAscope *in situ* hybridization

RNA *in situ* hybridization for mouse *Notch1* (404641-C1) and mouse *Lgr5 (312171-C2)* was performed according to the manufacturer’s instructions (Advanced Cell Diagnostics). Briefly, 7 μm paraformaldehyde-fixed, OCT-embedded frozen intestinal tumour sections were pretreated with heat in the retrieval reagent and protease III before hybridization with the target oligonucleotide probes. Preamplifier, amplifier and alkaline-phosphatase-labelled oligonucleotides were then hybridized sequentially. HRP signal was developed using TSA® Plus Fluorescein (Perkin Elmer) for the Notch1 probe and TSA® Plus Cyanine 5 (Perkin Elmer) for the LGR5 probe. Quality control was performed to assess RNA integrity with a probe specific to PolR2A RNA (320881) and for background staining with a probe specific to bacterial *dapB* RNA (320881). Specific fluorescent signal for *Notch1* and *Lgr5* was identified as green and far-red punctate dots, respectively. Samples were counterstained with anti-GFP antibody (chicken anti-GFP antibody Abcam ab13970, 1/1000) coupled to an anti-chicken Cyanine 3 secondary antibody (103-165-155 Immunotech SAS).

### smFISH quantification analysis

Single-molecule RNA FISH analysis and quantification were performed in a supervised semi-automatic manner: the perimeter of GFP positive cells was manually defined as Regions Of Interest (ROI) in selected Z sections using ImageJ, based on antibody immunofluorescence against membrane GFP. The same procedure was performed to draw the outline of GFP negative cells, using low signal background membrane staining. The Ilastik image classification and segmentation software (http://ilastik.org/) was used to segment the RNA FISH dots and was trained on several pictures for each of the two fluorescent channels (green and far-red) to classify punctate dots as either “Background” or “Dots” based on colours, intensities, edges and texture features. After Ilastik Batch processing, the segmented pictures were visually inspected for their consistency with the raw data. Enhancement of segmented pictures was performed by a unique erode function (whereby if a pixel is surrounded by background intensity pixels in a 3 × 3 matrix, it is eliminated from the analysis), allowing better separation of dots aggregates and suppression of small dots from low-intensity signals. The enhanced segmented pictures were then analysed using the “Particle analysis” function in ImageJ, with no size limit and circularity between 0.4 and 1.0 to obtain single dot ROI. To filter non-specific dots due to background fluorescence, the dot ROI in each channel was challenged against the corresponding dot ROI in the other fluorescent channel (green or far-red) and dots with more than 20% of overlapping surface, in both channels, were eliminated from the analysis. The number of dots per cell was obtained by counting all dots ROI having more than 50% of their surface included in each segmented cell perimeter defined previously. To clarify, a dot at the interface of 2 cells (which is quite often the case due to the large space occupied by the nucleus) was counted based on its surface distribution: if more than 50% of its surface was within a cell1, it would be <50% in cell2, thus it was counted uniquely in cell1.

### Cell cycle analysis by FACS

After dissociation, cells were fixed in 2% PFA for 20 minutes at 4 °C. Cells were washed twice in 1X PBS upon centrifugation at 400 g, 4 minutes at 4 °C, and staining proceeded. Cells were then incubated in pre-warmed 1X PBS Hoechst 33342 (1:100, stock at 10 mg/ml) solution for 20 minutes at 37 °C. FlowJo software was used for data analysis.

### Microscopy and image acquisition

For image acquisition, we used an upright confocal spinning disk microscope (Roper/Zeiss), equipped with a CoolSnap HQ2 camera and 405 nm, 491, 561 and 642 nm lasers. Images were captured using Metamorph. Image processing was performed using ImageJ software.

### Flow cytometry

Dissociated cells were incubated in Flow buffer (DMEM/F12, 5 mM EDTA, 1% BSA, 1% FBS and 10U/ml DNAse (D4263-5VL Sigma-Aldrich)) during 25 minutes at 4 °C with the following antibodies: EpCAM-PE/Cy7 (PE; R-Phycoerythrin, Cy7; Cyanine, 1:100, Biolegend clone G8.8), CD45-APC (APC; Allophycocyanin, 1:100, Biolegend clone 30-F11), CD31-APC (1:100, Biolegend clone MEC13.3), Ter-119-APC (1:100, Biolegend clone TER-119). To exclude non-viable cells, DAPI (1:1000, Sigma-Aldrich) was added. Cells were then washed and filtered directly into FACS tubes (40 µm strainer). Analysis was carried out on a FACS-LSRII and sorting on a FACS-Aria III (Becton Dickinson). For cell sorting, RLT lysis buffer supplemented with Beta-mercaptoethanol was used for RNA extraction (Qiagen). FlowJo software was used for data analysis.

### RNA extraction and qRT-PCR

RNA extraction was performed using RNAEasy Micro Qiagen kit according to the manufacturer’s instructions. Reverse transcription was performed using the SuperScript III First-Strand Synthesis System (ThermoFisher Scientific), according to manufacturer’s instructions. Random hexamer primers were used for reverse transcription. Real-time PCR quantification of gene expression was systematically performed in triplicate using SYBR Green I Master (Roche) on a ViiA 7 RT-PCR System (ThermoFisher Scientific). The efficiency of the primers used for real-time quantification (listed in Supplementary Table 2) was evaluated relatively to the slope obtained by the quantification of a standard curve, and the presence of single amplicons per primer pair, at the expected size, was checked on 2% agarose gels. Results were normalized to the expression of 18S, GAPDH and β-actin housekeeping genes and the relative expression was obtained with the −ΔΔCt method.

### AOM/DSS colon carcinogenesis experimental protocol

To induce colon tumours, we followed the protocol from Tanaka and colleagues^34^: Notch1-Cre^ERT2^/R26^mTmG^ mice ranging from 5 to 7 months of age were administered a single intraperitoneal injection of Azoxymethane (AOM, Sigma #A5486) followed by Dextran Sulfate Sodium (DSS, MP Biomedicals #160110) administration (3% in drinking water) one day after the AOM injection for 5 consecutive days. General health status and mouse body weight were monitored daily during and after treatment. To verify the presence of colon tumours, 2 mice were checked 1 month after the first cycle of DSS treatment, but no tumours were detected (only signs of inflammation). We administered another cycle of DSS (3% in drinking water) for 3 days and tumour formation was monitored by colonoscopy using a Karl-Storz endoscopic system.

### Affymetrix analyses and sample quality control

Transcriptome profiles were obtained using Affymetrix Mouse Gene ST 2.1 arrays. For each condition, three biological replicates were performed, by pooling intestinal adenomas and crypt fractions from more than 3 mice per replicate. cDNAs were synthesized from total RNA and hybridized by the Institut Curie Genomics Platform. Data normalization and analysis were performed using the R software (version 3.4.0). Raw data have been first normalized using the RMA method (R package Affymetrix 1.56.0)^44^, and annotated using the BrainArray *Mus musculus* mogene21st annotation library (19.0.2). Non-annotated or lowly expressed genes (<3.5) were discarded from the analysis.

Differential analyses were performed using the Limma package (3.34.0)^45^ and the following linear model: Y_ij_ = mu_i_ + T_t_ + G_g_ + (T*G)_tg_ + E_ijtg_ where Y_ij_ is the expression of gene *i* in sample *j*, T is the effect of sample type and G is the effect of GFP status. Results of the differential analysis have been corrected from multiple testing using the Benjamini-Hochberg procedure. Differentially expressed genes in the comparison of Normal GFP+ (Notch1+ ISCs) vs Tumour GFP+(Notch1+ tumour cells), and Tumour GFP+ vs Tumour GFP- (non-labelled tumour cells), have been defined using an adjusted p-value of 5% and a minimum log fold change of 0.5.

GSEA analysis^46^ was performed (with -nperm 1000 -permute gene_set -collapse false parameters) using the same expression matrix (v2.2.2), comparing GFP+ and GFP- samples^46^. Transcriptional signatures used for the analysis were extracted from the literature^30,32,33^. Normal GFP+ vs – signature have been defined using an adjusted p-value threshold to 25% and a minimum log fold change of 0.5.

Agilent data available from the literature^33^ (GSE37929) have been analysed using the Limma R package. Differentially expressed genes have been detected as previously with the same thresholds (adjusted p-values < 5% and a minimum log fold change of 0.5).

KEGG enrichment analysis has been performed with the ClusterProfiler R package (v3.6.0)^47^ to determine enriched pathways in Notch1-expressing tumour cells vs Notch1+ ISCs and Lgr5 Hi vs Low adenoma cells^33^. KEGG pathways with a q value < 0.25 were called significant. Figure 3h–j presents a list of manually selected pathways. The full list of significant pathways is available in Supplementary Table 1.

The concentration and integrity of RNA samples used for transcriptomic and qPCR analyses were evaluated with a Bioanalyzer using the RNA 600 Pico lab chip (Agilent) according to manufacturer’s instructions, as well as with the Tapestation system (Agilent). RNA integrity number (RIN) was confirmed to be higher than 8 for all the six samples. All transcriptomic experiments were performed using three biological replicates, consisting of cells pooled from at least 4 mice per replicate.

### Statistical analysis

Data are shown as Mean ± standard error of the mean (SEM). The Student’s t-test was used where applicable, with p < 0.05 considered as significant. Statistical analysis was performed using GraphPad Prism 7 (GraphPad Software, San Diego, CA, USA). n ≥ 3 independent experiments were used for statistical analysis, unless otherwise indicated.

## Supplementary information

Supplementary Data

Supplementary Table 1

Supplementary Table 2

## Data Availability

Affymetrix data sets have been deposited in the Gene Expression Omnibus (GEO) database with accession no. GSE111594. All additional relevant datas are available in the manuscript and its supplementary information files, or from the corresponding author upon request.
