# Supplementary material for: Lineage tracing of Notch1-expressing cells in intestinal tumours reveals a distinct population of cancer stem cells
Source: Sci Rep. 2019 Jan 29;9:888. doi: 10.1038/s41598-018-37301-3 (PMC6351556; doi:10.1038/s41598-018-37301-3)
Supplement: Supplementary file 1 — Supplementary Data [file 41598_2018_37301_MOESM1_ESM.pdf]

Supplementary Figures for article:

Lineage tracing of Notch1-expressing cells in intestinal tumours reveals a distinct population of cancer stem cells

Larissa Mourao[1,2,3], Guillaume Jacquemin[1,2], Mathilde Huyghe[1], Wojciech J. Nawrocki[4], Naoual Menssouri[1,5], Nicolas Servant[5,6] and Silvia Fre[1*]

Supplementary Figure 1. Gate strategies for FACS analysis

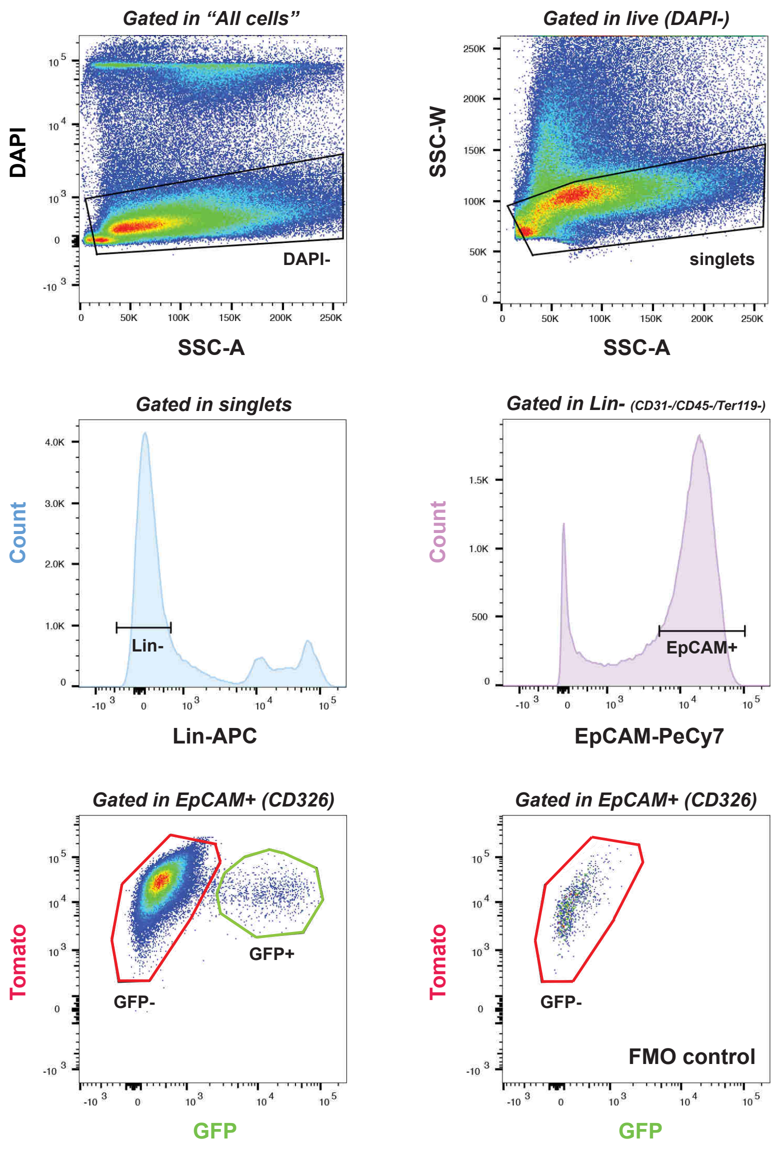

Supplementary Figure 1. General strategy for flow cytometric analysis and sorting of tumour epithelial cells. Representative dot plots of the FACS gating strategies used in this study. Live cells were gated in Dapi-/SSC-A. To remove cell aggregates, singlets were gated in SSC-W vs SSC-A. The sample was further analysed by selecting the Lin- population (Lin markers; CD31, CD45, Ter119), following for their uptake of the EpCAM (CD326) marker, resulting in the tumour epithelial cells fraction. Notch1-expressing cells were gated using the GFP/Tomato channels. FMO (fluorescence minus one) control was used to draw GFP+ gates.

Supplementary Figure 2. Quantification of clonal expansion by FACS analysis and immunofluorescence

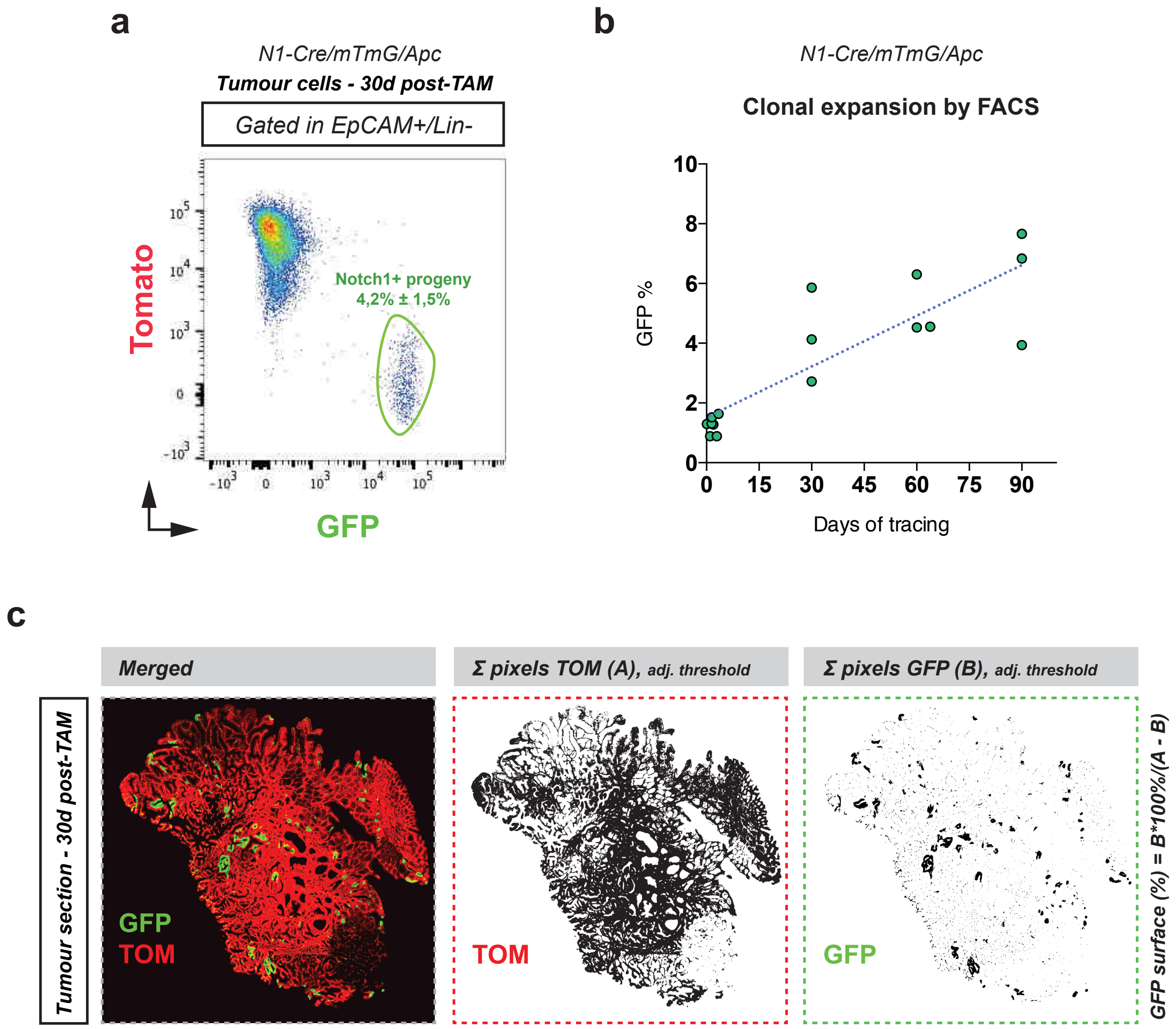

Supplementary Figure 2. Quantification of clonal expansion by FACS analysis and immunofluorescence. (a) Representative FACS dot plot of N1-Cre/R26^{mTmG}/Apc tumour cells analysed aftern a 30 days chase. The Notch1-derived progeny (GFP+/Tom-) no longer presents Tomato fluorescence. **(b)** Non-fitted FACS quantification of the clonal expansion of Notch1-expressing tumour cells. Each dot represents an independent biological replicate ($n \geq 3$ per time point). **(c)** Schematic representation of the quantified GFP+ area in intestinal tumours. Images show a section of a tumour exhibiting endogenous Tomato (TOM) fluorescence (in red) and clones derived from Notch1-expressing tumour cells displaying GFP fluorescence (in green). Split channels of binary composites for both TOM (a representing the number of pixels present within the TOM channel) and GFP (b; representing the number of pixels present within the GFP channel) were used to quantify the GFP+ area.
